# Young adults with OCD and their experiences of the school-to-work or university transition: an explorative qualitative study

**DOI:** 10.1186/s12888-026-08714-6

**Published:** 2026-10-13

**Authors:** Åsa Andersén, Iman Alaie, Beatrice Carpentsier, Magnus Helgesson, Lisa Holmlund

**Affiliations:** 1 Division of Public Health, Working Life and Rehabilitation, Department of Public Health and Caring Sciences, Uppsala University, Box 564, Uppsala, SE-751 22 Sweden; 2 Division of Clinical Psychology, Department of Psychology, Uppsala University, Uppsala, Sweden; 3 Division of Insurance Medicine, Department of Clinical Neuroscience, Karolinska Institutet, Solna, Sweden; 4 Division of Occupational Therapy, Department of Neurobiology, Care Sciences and Society, Karolinska Institutet, Huddinge, Sweden

**Keywords:** School-to-work or school-to-university transition, Employment, Obsessive compulsive disorder, Young adult, Thematic analysis, Qualitative

## Abstract

**Background:**

The school-to-work or school-to-university transition (STWT) can be a challenging process for young adults, especially for those suffering from mental disorders such as obsessive-compulsive disorder (OCD). While OCD is a relatively common disorder, little is known about how young adults with OCD experience the STWT process. A better understanding of the conditions influencing STWT may deepen knowledge relevant to the development and implementation of interventions aiming at promoting labour market inclusion for this population. This study aimed to explore how young adults aged 19–29 years living with OCD experience the STWT and which factors they perceive as challenging or supportive during this process.

**Materials and methods:**

The study employed an explorative qualitative design. Data were collected through semi-structured individual interviews with 21 participants (14 females and 7 males) diagnosed with OCD, with a mean age of 23.0 years at the time of the interviews. Data were analysed using reflexive thematic analysis.

**Findings:**

Two themes were generated: (1) Navigating pathways into the labour market through studies or work while managing health and (2) Navigating pathways into the labour market through societal support. The analysis illustrates how OCD symptoms, as well as organisational and societal conditions, could create challenges that affected and, in some cases, delayed the STWT process. Participants struggled to manage these challenges while also prioritising their health. Throughout this process, they developed their own strategies and used various forms of support.

**Conclusion:**

The findings suggest a potential interplay between OCD symptoms and engagement in education and employment during the STWT process. Increased OCD symptoms, worsening mental health, and unmet individual needs may contribute to delay or interrupt the transition. Individual coping strategies, pedagogical, workplace-related, social and societal support may help young adults overcome challenges while maintaining their mental health and well-being. The findings emphasise the need for flexible, individualised support and coordinated efforts among relevant stakeholders. Healthcare services may address both symptom management and the new contexts and challenges embedded in the transition. Increased awareness of OCD, together with sustainable and supportive study and work environments, is important for improving inclusion in working life among young adults with OCD.

**Supplementary Information:**

The online version contains supplementary material available at https://doi.org/10.1186/s12888-026-08714-6.

## Background

Employment is considered essential for societal participation [1]. It provides financial security [2], contributes to the development of a work identity and social status [3], and promotes a sense of being active and contributing to society [4]. In this context, the transition to working life and associated work outcomes is important for young adults. The period, during which young people are expected to enter the labour market or pursue post-secondary education, e.g. university studies or vocational training (the school-to-work and school-to-university transition, STWT) represents a critical phase that can significantly influence their health and well-being [5]. Young adults who participate in post-secondary education, employment or vocational training programmes generally report better health and well-being than those who are unemployed [6, 7].

The STWT constitutes an important foundation for future occupational development and usually occurs alongside other important life events and social processes. These may include changes in relationships and living arrangements, such as moving out of the parental home and establishing independent housing [8], becoming more independent, and shaping one’s own life [6]. At the same time, individuals encounter new contexts and conditions as they enter work or begin post-secondary education [8]. Over time, the STWT process has become more varied and prolonged, partly due to shifts in the labour market from more traditional and stable forms of employment to more uncertain conditions. Consequently, young adults may experience repeated periods of unemployment but also more frequent transitions back into education and work-based training [8].

Additionally, the STWT is influenced by factors such as educational level, geographical location, and individual skills [8, 9]. To meet labour market demands and contribute to productivity, individuals often need to develop new skills [10]. Unemployment during this period may pose risks for future labour market participation, including an increased risk of subsequent periods of unemployment and poorer employment opportunities [11]. For instance, unemployment can hinder the development of work‑related skills, which may in turn affect future employability [8]. Moreover, periods of unemployment during the STWT process can be a source of stress for young people [8].

Obsessive-compulsive disorder (OCD) is a prevalent, multifaceted and often underdiagnosed mental disorder [12], characterised by intrusive, recurrent, persistent and unwanted thoughts, images or urges (i.e. obsessions) [13]. It typically also involves repetitive behaviours and/or mental acts (i.e. compulsions). In response to obsessions, individuals often feel compelled to perform specific compulsive behaviours or to ignore, suppress or neutralise them to alleviate anxiety and distress [13]. The condition can include obsessions and compulsions across many domains, for example, contamination or symmetry [14], and may manifest in various ways over time [12]. The lifetime prevalence of OCD is approximately 2.3% [15]. The median age of onset of OCD is 19 years [15, 16], and is similar in both sexes, with the highest likelihood of onset between the ages of 17 and 22 [16]. A substantial delay between OCD symptom onset and diagnosis by a clinician has been reported, with a mean delay of 11 years [17]. Among individuals younger than 27 years, the mean time from first experiencing clinically significant OCD symptoms to receiving a diagnosis was estimated at four years [17]. Common comorbid conditions include anxiety disorders, mood disorders, impulse-control disorders, and substance use disorders [15]. OCD can be time-consuming and significantly affect key aspects of life, including occupational [18] and social functioning [13, 19].

In Sweden, young adults with disabilities, including mental disorders, often face difficulties in establishing themselves in working life [20, 21]. Mental disorders are also the most common cause of sickness absence among individuals aged 19–29 years [22]. Furthermore, 70% of those granted temporary compensation for reduced work capability, have a psychiatric diagnosis [23]. Similar patterns are observed in other European countries, including Norway, the United Kingdom and Denmark, where mental and behavioural disorders constitute the most common diagnostic group among those receiving disability benefits in this age group [24], although social security systems vary across countries. Previous Swedish national register-based studies have investigated clinically diagnosed OCD in relation to educational attainment and labour market marginalisation. One study found that individuals with OCD were less likely than controls to attain each level of education, from primary school to post-graduate education, particularly among those diagnosed before the age of 18 years [25]. Another study reported increased risks of long-term sickness absence and unemployment while the likelihood of receiving disability pension (i.e., social insurance compensation for individuals aged 30 to 64 who are assessed as having a permanent full- or part-time work disability, or temporary compensation for young individuals, aged 19–29 years, in case of reduced work capability or prolonged schooling) was 16 times higher among individuals with OCD compared with the general population [26].

In Sweden, the Social Insurance Agency (SIA) administers various forms of compensation within the social insurance system and is responsible for assessing eligibility for compensation for work disability due to illness or disability [27]. The SIA also assesses the need for vocational rehabilitation and is responsible for coordinating these efforts. In addition, the Public Employment Service (PES) provides support to job seekers and may offer targeted interventions to individuals with reduced work ability [28].

In summary, the STWT can be a challenging process for young adults, especially for those suffering from mental disorders such as OCD. Although OCD is a relatively common disorder associated with lower educational attainment, poorer prospects of entering the labour market and an increased risk of long-term sickness absence and disability pension, there is still limited research on targeted interventions and support programmes that may facilitate the STWT process for young adults with OCD. A better understanding of affected young adults’ lived experiences of the STWT process may deepen knowledge and enable the development and implementation of interventions aimed at promoting labour market inclusion for this population. Therefore, this study aimed to explore how young adults aged 19–29 with OCD experience the STWT period.

## Methodology

### Study design and setting

This study employed a qualitative design with an explorative and constructivist approach. Data were collected through semi-structured, in-depth interviews and analysed using inductive, reflexive thematic analysis following the six phases described by Braun and Clarke [29]. The study is part of a larger research project exploring facilitators and barriers to entering the labour market and achieving sustainable employment among young adults aged 19–29 years with OCD.

The project is conducted in collaboration with the Swedish OCD Association, a nationwide organisation that supports individuals with OCD and related disorders, as well as their family members. A reference group was established for the project, comprising representatives from the Swedish OCD Association and young adults with lived experience of OCD. The research team comprised three women and two men with diverse professional backgrounds and disciplinary expertise.

The authors form an interdisciplinary research group based at Swedish universities with expertise in worker health, including labour market participation and sickness absence among people with mental disorders. Å.A. holds a PhD in medical sciences, is a registered nurse and public health scientist, and has clinical experience in healthcare. I.A. holds a PhD in medical sciences, is a licensed psychologist with clinical experience in child and adolescent psychiatry. B.C. holds a MSc and is a registered psychiatric nurse. M.H. holds a PhD in medical sciences and has a background in public economics and political science. L.H. holds a PhD in medical sciences and is a registered occupational therapist with clinical experience in healthcare. Å.A., L.H. and M.H. have no clinical expertise in OCD. The first and last authors have extensive experience in qualitative research and reflexive thematic analysis. This study followed the Reflexive Thematic Analysis Reporting Guidelines (RTARG) [30].

### Participants and recruitment

In the first phase, participants were recruited via Uppsala University’s website and community partners, including the Swedish OCD Association. Due to a low recruitment rate, a second recruitment phase was initiated via social media (Instagram and Facebook). Eligibility criteria were: being aged 19–29 years, having a clinical diagnosis of OCD, having completed upper secondary education/other education (a full degree/certificate was not required), being unemployed, studying or having started a job within the past 12 months, and being able to communicate in Swedish.

A total of 84 individuals expressed an interest in participating in two related studies, of whom 42 were initially considered suitable for the present study according to the inclusion criteria. A strategic sampling approach was used to recruit participants who shared the experience of being in the STWT period and to ensure at breadth of experiences thorough variation in participant characteristics, including gender, ages, primary STWT status (employed, unemployed or studying), and region of residence. Therefore, the 42 individuals were contacted by a research assistant via telephone, who provided further information about the study and collected background information. They also received written study information and were offered a follow-up telephone call if they had any further questions. Of the 42 individuals, four declined to participate, five did not meet the eligibility criteria, one did not respond to the telephone call, six did not return the signed consent form, and five were not included due to the strategic recruitment approach.

In total, 21 individuals were recruited. After providing written informed consent, all participants were contacted by the first author (20) or the last author (1) to schedule an interview and choose their preferred interview mode (telephone or Zoom). The decision to offer participants the choice of a telephone or Zoom interview was made in consultation with the reference group. Providing two options for conducting the interview aimed to facilitate participation from individuals living in different regions of Sweden and to ensure that participants could select the format in which they felt most comfortable and secure. Zoom interviews could also be conducted without video if preferred. To establish rapport, the authors informed participants about the interview procedure and the topics to be discussed. Participants were also provided with information about the study’s purpose, confidentiality, voluntary participation, and their right to withdraw from the study at any time.

Of the 21 participants, 14 were women. The mean age was 23.0 years. At the time of the interviews, 12 participants were studying full-time or part-time, two were employed part-time, and seven were unemployed, of whom five were registered with the PES. For detailed characteristics of the participants, see Table 1.

### Dataset generation

Interviews were conducted by the first (*n* = 20) and last (*n* = 1) authors, both of whom have extensive experience in conducting qualitative interviews. The interviews were conducted between June 2023 and March 2024 and lasted between 38 and 86 min. Eleven interviews were conducted by telephone and ten via Zoom.

A semi-structured interview guide was developed for this study in collaboration with the reference group (see Supplementary File 1). The guide covered participants’ experiences of everyday life with OCD; their experiences during upper secondary education (to contextualise participants’ situations); the transition from upper secondary education to post-secondary education, employment or other activities; and perceived barriers and facilitators to entering and engaging in employment or education from individual, organisational and societal perspectives. Although secondary school experiences were not the primary focus of the analysis, aspects of these experiences that were relevant to the STWT were included to get a better understanding of the STWT process. Examples of questions included: *What does a typical day look like currently? How does OCD manifest itself in your daily life? What expectations did you have about working or studying after completing upper secondary education? Can you describe how you think about working? How did you view your opportunities to work after upper secondary education?* The complete interview guide is presented in Supplementary File 1. Participants were asked to elaborate on their responses and provide examples of situations or events to enable an in-depth understanding of their experiences and context. Two pilot interviews were conducted, resulting in minor revisions to the interview guide. Field notes were written after each interview. All interviews were audio-recorded and transcribed verbatim by the third author (B.C.).

**Table 1:** Sociodemographic characteristics of the participants

Demographic characteristics	*n* = 21
**Gender**, *n*	
Female	14
Male	7
**Age**, *years*, mean, (range)	23.0 (19–28)
**Educational level**	
Upper secondary education	16
Post-secondary education/University	5
**STWT states (**primary)	
Studies, full‑time /part‑time	8/4
Work, full-time/part-time	0/2
Unemployed (of whom 5 were registered with the PES^1^)	7
**Living arrangements** ^**2**^	
With parent/parents	7
Alone	4
With a partner	9
**Children living at home** ^**3**^	2
**Current health care contact**, yes	10

Data are based on the interview time point

PES: Public Employment Service

^1, 2^ Missing data for one participant

^3^ These data emerged from the interviews and were not collected through questions specifically asked of all participants

### Data analysis

The data were analysed following the six-phase process for reflexive thematic analysis, as suggested by Braun and Clarke [29]. The method is theoretically flexible and widely used for analysing qualitative data. It provides a structured, open, and interactive analytical process suitable for exploring deep and nuanced meanings and understanding patterns within data in their specific context [29]. NVivo 14 software was used for data management.

Initially, the first author listened to the original recordings and read the transcripts repeatedly to familiarise herself with the dataset and to check for accuracy. After reading the transcripts, reflections and a summary of the interviewee’s story were documented, followed by analytical discussions between Å.A., B.C. and L.H. During the initial coding phase, data segments relevant to the research question and considered important were marked. The last author independently examined a selection of transcripts and coded three interviews. The authors then discussed similarities and differences in their coding and interpretations, as well as their underlying assumptions to deepen their analysis and broaden interpretative perspectives. To further enhance analytical rigour, the first and third authors refined the coding across the dataset, resulting in codes being added, merged, renamed or removed. Examples of codes included *Considerations for future work* and *Perceptions of facilitating factors for participation in education*. To develop initial themes, analytical discussions based on reflections helped identify patterns of meaning and develop candidate themes by mapping ideas derived from the initial coding. These discussions were guided by questions such as: What stayed with you when reading the transcript? Are there shared experiences across the interviews? Ideas were critically discussed between the first and last authors in relation to participants’ attempts to enter employment or engage in studies and how their perceptions of how these processes were related to their OCD symptoms.

This analytic mapping reflected an overarching narrative of participants’ efforts to establish themselves in the labour market, for example, through pursuing studies, undertaken temporary employment, and seeking access to societal support. In these reflexive discussions, the authors drew on their understanding of work ability/disability as relational concepts [31], thereby positioning participants’ individual experiences and efforts in relation to organisational and societal contexts. Overall, the analysis followed an iterative process, in which themes were continuously and reflexively discussed between authors, situated contextually and checked against both codes and the dataset. This process aimed to develop a nuanced analytical understanding in relation to the study’s aim.

### Analysis

The STWT was an ongoing process in which young adults explored possible pathways through work and education. These pathways were navigated within unfamiliar work and study environments and systems (such as the public employment service), while participants also managed OCD symptoms and related health concerns. In this process, obsessions and compulsions consumed time, energy and attention, posing major challenges to everyday life, including entering and pursuing education and employment. Lack of energy made it harder to resist symptoms, which could be exhausting and limiting, potentially affecting mood negatively. The severity and nature of symptoms varied over time. During the STWT process, stress or exposure to new situations, such as moving away from home, could further affect well-being, exacerbate symptoms or even alter the presentation of OCD symptoms. Overall, the analysis suggests that the STWT for young adults with OCD involves constant navigation of different pathways through organisational and societal contexts with a persistent chance of interruption or delay depending on the challenges encountered and the support received. Two themes, along with associated subthemes, were generated in the analysis, illustrating participants’ use of individual strategies and various forms of support to navigate, manage and address challenges, as well as to maintain well-being during the STWT process (see Table 2 for an overview of the themes, subthemes and characteristics of content).

**Table 2:** Themes, subthemes and characteristics of content

Theme	Subtheme	Characteristics of the subthemes
Navigating pathways into the labour market through studies or work while managing health	*Challenging and supportive conditions for study participation* *Challenging and supportive conditions for entering work* *Strategies and support to overcome challenges and maintain health*	OCD symptoms and deteriorating mental health could complicate the study process with the potential to delay the STWT process. Factors that facilitated study participation included, e.g. flexible forms of education (e.g. distance learning), pedagogical accommodations (e.g. mentoring support, studying in smaller groups, audio textbook service, etc.), and adaptation of examination (oral or extended time). Entering work involved confronting new situations and workplace demands. This could exacerbate OCD symptoms and mental health difficulties, thus complicating the STWT process and increasing the eventuality of delays due to sick leave or even discontinuation of employment. In contrast, work could also serve as a distraction from OCD symptoms. Facilitating factors included prior positive work experiences, supportive workplace and work environments that focused on individuals’ strengths rather than their difficulties. To manage the STWT and cope with OCD symptoms, participants used various strategies and sources of support. These included resisting compulsive behaviours during studies or work, maintaining daily routines and planning ahead. To promote mental health and well-being, participants described taking regular breaks, engaging in physical or creative activities. Factors perceived to facilitate future work participation included adjusted working hours, less stressful jobs and flexible working hours. Support from family, partners and friends was also important in managing the STWT and reducing stress and OCD symptoms.
Navigating pathways into the labour market through societal support	*Dissonance between support and individuals’ needs* *Healthcare support as a valuable resource throughout the STWT* *Stigma and lack of knowledge as barriers to openness*	A lack of communication between actors and individually tailored support could delay the STWT process. Being referred within and between organisations and managing multiple contacts were perceived as stressful and negatively impact mental well-being. Experiences of support from the PES varied, with some participants viewing it as a possible way forward to employment. Contact with healthcare during the STWT was described as important for managing OCD symptoms, developing coping strategies for different situations (e.g. in the workplace) and maintaining well-being. This support could include medication and/or psychological contact. Participants described stigma and limited awareness of OCD in society as barriers to disclosing their diagnosis. Concerns included fear of rejection during recruitment, being replaced by another applicant, or being seen as different. At the same time, not disclosing the diagnosis could mean missed opportunities for support. Some participants considered disclosing their OCD diagnosis, while others did not. Among those who had disclosed at the workplace, this had meant increased support and understanding.

### Theme 1: Navigating pathways into the labour market through studies or work while managing health

The first theme focuses on how OCD symptoms and mental health challenges could hinder participants’ engagement in new work and study environments, with the potential to delay the STWT process. Throughout the transition, participants sought strategies and supportive conditions to handle these challenges while also maintaining their health.

#### Challenging and supportive conditions for study participation

OCD symptoms and deteriorating mental health complicated participants’ engagement in education. However, the type of educational programme and pedagogical support could facilitate their participation in studies.

Most participants had completed upper secondary education; however, many had experienced difficulties during their studies due to OCD symptoms or comorbid mental health conditions (e.g. depression or anxiety), which adversely affected their well-being. Many participants were diagnosed with OCD during upper secondary education, while several of those diagnosed earlier, reported a worsening of their symptoms during this period. The consequence of not graduating from upper secondary education was having to catch up on their studies later in life to create opportunities for further education or employment. As one participant explained:

*… I felt like*, “*Oh shit. I’ve done three years of upper secondary school and I’m still not finished and now I have to do another three years at folk high school”* (P10, woman).

Participants described how interrupted education, together with limited opportunities to gain work experience, made it more difficult to establish themselves in the labour market. As one participant described:

*I haven’t exactly had an easy time on the labour market or as a student either. I have been out of education [spent time at home] for a very long time and basically already after compulsory school*,* so after Year 9*,* I sort of left school and stayed at home. So I didn’t… I didn’t catch up on compulsory school until I was 23*,* I think.* (P17, man)

As illustrated by the quotes, delays in the STWT were linked to difficulties completing education and the need to improve unsatisfactory or incomplete grades after upper secondary education. Similarly, worsening OCD symptoms or deteriorating mental health due to comorbid conditions, such as anxiety or depression, during post-secondary education (e.g. university studies) could lead to a negative spiral. When studies had to be paused or interrupted, participants described how this delayed their educational progression and, in turn, postponed their transition to working life.

Participants described how OCD symptoms manifested in educational settings in different ways. This included fears of making mistakes or being negatively assessed by others. Some participants experienced a strong pressure to perform well or achieve perfection. Hence, they found it challenging to initiate study tasks, arrive at school on time, or complete and submit assignments on time. Additional challenges included getting stuck on a letter or words, repeatedly rewriting or rereading a text until it felt ‘just right’, or focusing on neat handwriting instead of the content of the written work. Two participants shared:

*…but I’m constantly thinking*,* “I should start studying now” or “I should do this and that”*,* but it’s like I just go*,* “No*,* it has to be perfect first and then I can start”.* (P9, woman)

*I tend to have difficulties finishing my tasks. I struggle to get to school and to attend meetings I’m supposed to attend*,* and I tend not to study as much as I need to in order to pass the course.* (P7, man)

For those needed to catch up on their studies later in life, subsequent studies in municipal adult education (Komvux) and folk high schools (Folkhögskolor) were often experienced as more successful. As one participant explained:

…*if I were to say one thing I’ve learned*,* it would be that I can really recommend Komvux and that form of study*,* where we were only in school a couple of days a week. We basically worked at our own pace. The demands weren’t so high.* (P17, man)

For some participants, remote learning made it easier to study, for example, by increasing flexibility to organise their time, reducing the need to travel. Most participants engaged in education were aware of available pedagogical support, including access to audio textbooks, adaptation of examination (e.g. oral examinations or extended time) and mentoring to support the planning and organisation of their studies. One participant described:

*…then the study support helped me by giving me some tips and advice*,* and suggestions on how to structure a scientific text and how to set it out*,* and that made things much easier.* (P11, woman)

Other pedagogical accommodations included access to individual study spaces and opportunities to study in smaller groups. Participants described how these adjustments, together with flexibility they offered, facilitated study participation, supporting their engagement in education.

#### Challenging and supportive conditions for entering work

Entering work involved confronting new situations and workplace demands. This could exacerbate OCD symptoms and mental health difficulties, thus complicating the STWT process and increasing the eventuality of delays due to sick leave or discontinuation of employment. However, there were also factors perceived as facilitating work-entry.

Several participants had work experience in various working conditions and for varying durations. One key challenge in entering employment was hourly or short-notice work, which created stress when participants were unable to maintain routines due to unpredictable scheduling. Other challenges included concerns related to hygiene and perfectionism. Work engagement often meant confrontation with various stressors, making it difficult to focus due to obsessions and compulsive rituals, e.g. double-checking, which contributed to fatigue.

OCD symptoms also impacted work performance, including needing more time to complete work tasks, making it challenging to meet colleagues’ expectations and work-related goals while simultaneously managing obsessions and compulsions. In contrast, for some participants, the work served as a distraction that kept obsessions in check or limited compulsive behaviours by keeping them occupied in a workplace surrounded by others.

*Yes*,* it’s like there isn’t really space there to keep… to keep obsessing and doing OCD-type things. At the same time*,* I can also get into a “flow” where there isn’t so much compulsion if I don’t have time to think.* (P19, man)

In some cases, a poor work environment (e.g. long work shifts with limited recovery time), in combination with OCD symptoms, was described as negatively affecting participants’ health. For some, OCD symptoms became so difficult to manage that they chose to end their work, and for others, the situation eventually resulted in sick leave. There were also cases where employers terminated or chose not to extend temporary or hourly contracts, e.g. due to repeated sickness absence or concerns that the employee was unwell.

*I had temporary employment from… yes*,* what was it*,* February to maybe June (mm) and I knew I had it. Then as it was coming to an end and I was going to ask whether I could get an extension*,* the manager said that it wasn’t possible because I was ill too often.* (P4, woman)

Difficulties in entering or remaining in employment were described as negatively affecting self-image and health. In such situations, participants often described trying different pathways to find suitable work, considering their well-being and OCD. Conversely, previous experiences of completing work tasks, e.g. summer jobs, were reported to strengthen belief in their ability to manage future employment. As one participant described: ‘*…I really want a job and I’ve been able to have jobs before because I’ve done summer work in the past*,* basically’* (P1, woman). A workplace that offered support and focused on strengths rather than difficulties was perceived to increase participants’ capacity and perquisites for managing work.

#### Strategies and support to overcome challenges and maintain health

To manage the transition into studies or employment, participants sought social support and applied various strategies to cope with challenges associated with OCD, trying to maintain mental health and well-being throughout this process.

Participants described developing strategies to manage difficulties in work and study contexts, reduce OCD symptoms, and maintain their health and well-being. These included adopting personal rules to resist compulsive behaviours. Maintaining daily routines was considered important as it provided predictability and a sense of control. Structure, clarity and predictability were emphasised as important enabling factors for participation in both study and work.

*Yes*,* what really helps me a lot is that I really like knowing exactly what’s going to happen in detail*,* like “OK*,* I’ll arrive on this day and I’ll be here for this long. I’ll have lunch at this time and I’ll go home at this time*,* and this is the task that needs to be done and these are the people I’ll meet’.* (P10, woman)

Engagement in physical activity or music could help participants to focus during the transition, as it alleviated anxiety during stressful periods. Living with OCD symptoms was considered energy-consuming, making recovery time and breaks between activities important. Strategies for self-care and maintaining well-being included reducing sensory input, such as sounds and other stimuli, to restore energy, engaging in activities, or undertaking enjoyable tasks. Participants also commonly engaged in creative pursuits, e.g. painting, to distract from obsessions and anxiety and, for some, to reduce passivity.

*…if I notice that I get stuck in a loop of obsessive thoughts*,* they just come and it doesn’t stop then … I usually try to go for a walk*,* or do something I enjoy*,* or I just interrupt what I’m doing at that moment.* (P15, woman)

Participants emphasised the importance of balancing work and recovery, and some believed that working hours would need to be reduced to part-time in order to support their well-being either in current or future employment. Some participants also reflected on factors they perceived as potentially facilitating future workforce participation, taking their OCD into account, e.g. reduced job demands or flexible working hours.

For most participants, support from family, partners, or friends was central. This support meant having someone by their side whom they could turn to for help, draw motivation from, and engage in open dialogue with.

*My mum. She has been there since I was little and has supported me through everything*,* and my boyfriend as well. Those two have… my boyfriend*,* my mum and my closest friend. Those three have supported me through everything. They’ve been there through thick and thin.* (P16, woman)

Some participants expressed that talking with relatives or friends about OCD or other difficult or burdensome experiences could help reduce stress or alleviate symptoms. This social support was important in everyday life, and some participants described it more explicitly in relation to participation in studies and/or entry into employment. Besides emotional support, family and friends provided practical assistance, such as completing applications for post-secondary education or requesting pedagogical accommodations. Parents also supported communication with authorities or healthcare providers when necessary. Furthermore, family members or partners could identify when OCD symptoms had worsened and help raise awareness of these changes. Although most participants reported receiving support and understanding from family or friends, the extent of this support varied; for some, it was lacking, leaving them alone to cope with their situation.

### Theme 2: Navigating pathways into the labour market through societal support

The second theme describes participants’ experiences of societal support during the transition into work. It illustrates how limited coordination between organisations and lack of individualised support could delay the STWT process. Perceived stigma and limited awareness of OCD were described as barriers to disclosing the condition, potentially resulting in unmet support needs. The theme also highlights the importance of healthcare support throughout the STWT process.

#### Dissonance between support and individuals’ needs

To facilitate labour market entry, many participants emphasised the importance of personalised support and effective communication between the organisations involved. When these conditions were lacking, participants described increased stress and a greater eventuality of delays in the STWT process.

Managing contacts with different organisations, such as the SIA and the PES, could be challenging. Participants described how changing contact persons, being referred between or within organisations to obtain information about e.g. benefits or being passed between healthcare providers while trying to access appropriate care often required them to repeatedly explain their situation. These experiences were perceived as stressful and negatively affected their mental health.

I feel it’s been very unclear information from there (mm), and it has given me a lot of anxiety, basically… The person I had as my contact there couldn’t give me the right answers and then… I had to stand in a queue for 40 min to get through to the Public Employment Service and speak to someone else, and then I was sent on to another person, so it was just… there was so much back and forth like that. (P1, woman)

Lack of communication and coordination between healthcare services, the SIA, the PES, the municipality, and/or the individual could delay access to interventions and appropriate support. Participants also described how poor communication, for example, between the PES and employers, meant that individual needs were not always recognised or accommodated. In addition, when regulations did not consider participants’ health conditions, the conditions for job training/work engagement were negatively affected, sometimes limiting the possibility of continued participation.

*…so because they kept adding more and more hours [during work-training] even though I felt like I hardly had the energy*,* it didn’t really work out. So in the end it was basically that I either quit*,* or I… I got in touch and said I couldn’t come into work that day*,* and then I ended up doing that more and more*,* and eventually it became like it just wasn’t working*,* and then I had to leave…* (P18, man).

Difficulties in obtaining individualised and appropriate support meant that participants often had to explore new pathways (with or without support from the SIA/PES) to identify suitable employment and paths to get there. Some participants were waiting for or had received support from the PES and viewed this as a possible path forward. There were also experiences where job training went well and led to employment, or to pursuing further education in preparation for a specific profession.

#### Healthcare support as a valuable resource throughout the STWT

Contact with healthcare services during the STWT was described as an important source of support for managing OCD symptoms, developing strategies to cope with different situations (e.g. in the workplace), and maintaining well-being throughout the transitional period.

Most participants had received treatment for OCD, including pharmacological treatment, psychological treatment, or both, with varying outcomes. Participants’ contact with healthcare services also varied: some had regular contact, whereas others did not. Many described having an ongoing contact with a psychologist as particularly valuable, providing someone to talk to and support in managing everyday challenges. As one participant described:

*… going to see the psychologist is basically the only real lifeline I have*,* really. I feel so much better just from talking about it. It doesn’t always have to be exercises or anything like that—just being able to talk about it and learning about OCD and things like that helps me enormously.* (P3, woman)

Talking about OCD and aspects perceived as difficult was described as helpful. In addition, psychological support could assist participants in finding strategies and guidance to manage OCD symptoms in different situations, for example, in the workplace.

*Like*,* if I ran into a problem one day (during work training)*,* it wasn’t a very long day*,* so I could ask the psychologist*,* “What do I do when this happens next time?” And then I started to understand more and more how I should do things and think…* (P4, woman).

Some participants reported receiving support from healthcare services, such as medication and psychological therapy, during their studies due to worsening mental health. Some were awaiting such support, while others wished to receive it in the future, to facilitate their participation in studies and/or entry into the workforce, and to promote their well-being.

#### Stigma and lack of knowledge as barriers to openness

Participants described experiences of stigma and limited knowledge and understanding of OCD in society, including in workplace and educational settings. For many, this led them to refrain from disclosing their diagnosis in order to avoid stigma or discrimination. However, this also meant potentially missing opportunities for support.

Fear of stigma or experiences of self-stigma were common reasons for not disclosing an OCD diagnosis in workplace or educational settings. Instead, participants shared that it was something only the family, partners or close friends knew. They also described encountering misunderstandings and preconceived notions about OCD, including expectations that people with OCD behave in a certain way.

*Yes*,* because I know that… 90% of the people whom I tell that I have OCD*,* the first thing they say is*,* “Oh*,* so you want everything to be neat and tidy”*,* and I say*,* “No*,* my room is chaos”’* (P16, woman).

OCD was also described as something people makes jokes about, as one participant stated:

*… it’s not taken seriously*,* and I think that’s something that needs to change in universities and in working life as well.* (P13, man)

Such experiences could contribute to a sense that the diagnosis and the challenges associated with OCD were not understood or taken seriously.

Although some participants wished to disclose their diagnosis because it was seen as increasing the possibilities for support, they were often uncertain about how it would be received. Among those in education, experiences varied: some had disclosed their diagnosis to fellow students and teaching staff, while others reasoned that if it proved to be an issue, they would share it. Some participants reported that they had not, or would not, disclose their diagnosis when applying for or starting a job because they feared being rejected or replaced by someone else.

*I think I would like to be open about having OCD in order to get some support in working life*,* but at the same time I’m afraid it would reduce my chances of getting a job*,* if people knew I had OCD and saw it as… well*,* something that made them not want to hire me. So*,* I feel a bit conflicted about it.* (P15, woman)

At the same time, some participants intended to disclose their diagnosis, and others had positive experiences of doing so in the workplace (for example, to managers and/or colleagues). Disclosure was perceived to increase support and understanding. For some, this led to increased self-confidence. Others preferred to disclose their diagnosis only after they had demonstrated they were qualified for their work. Many participants expressed a need for increased knowledge and awareness of OCD and mental illness in general within educational settings, workplaces and society at large, as a measure to ease the STWT process for young adults.

## Discussion

This study aimed to explore how young adults with OCD experience the STWT period and the factors they perceive as challenging or supportive during this process. The analysis suggests a potential interplay between OCD symptoms, mental health and the new situations and demands associated with the STWT. Worsening OCD symptoms and mental health could contribute to delays in the STWT. Participants navigated various pathways through education, employment and unemployment, using individual strategies and drawing on pedagogical, workplace, social and societal support to move forward while also maintaining their mental health and well-being. However, societal support could sometimes be difficult to access, be fragmented, or inconsistent. At the same time, refraining from disclosing the diagnosis could reduce opportunities to receive support and accommodations in educational and workplace settings. Taken together, the findings suggest that individual, organisational and societal context may influence participants’ STWT processes.

The findings also showed that, for some participants, OCD symptoms or worsening mental health due to comorbidity (e.g. depression or anxiety) contributed to difficulties during their education. For some this had resulted in incomplete upper secondary education, or in post-secondary education being interrupted or put on hold, thereby delaying the transition into working life. These findings align with previous research, where individuals with OCD described that education and career had been affected and life course goals delayed because of the OCD [32].

Given that the median age of onset of OCD is 19 years [15, 16], with the most significant development occurring between the ages of 17 and 22 [16], many young people receive their diagnosis shortly before or during the expected STWT period. This development stage can be stressful in and of itself [8], requiring young adults to acquire new skills and competencies for autonomous functioning [8, 10]. Together, these factors may place young adults in a particularly vulnerable position during the STWT. Given the gap between OCD symptom onset and diagnosis [17], improving the early detection of OCD within healthcare is essential to ensure that individuals receive appropriate treatment and support at an earlier stage, which may improve well-being and facilitate the STWT.

In previous research, individuals with OCD who did not complete upper secondary education were often able to find other pathways to qualify for university, for example, through adult education [25], which our study confirms. Moreover, research suggests that the impact of OCD symptoms on functioning may improve over time. This may reflect that some individuals become better at managing their symptoms with increasing experience and age, or that access to effective treatment enables them to resume their education [25].

Several participants who had experienced difficulties in education earlier in life found subsequent studies in municipal adult education or folk high schools to be more manageable, and many also reported positive experiences of post-secondary education. Participants highlighted the value of pedagogical accommodations, e.g. adapted examinations, audio textbook services, and mentoring support. These findings suggest that, despite earlier difficulties, later/other forms of education may be more successful and therefore be a potential pathway to employment. An important implication is the need for clear and transparent processes for obtaining pedagogical accommodations throughout the education system.

The findings showed that entering employment involved confronting new situations and stressors, which could influence OCD symptoms and well-being. Worsening OCD symptoms and deteriorating mental health could lead to sick leave or job loss, creating a negative spiral that could further exacerbated both mental health difficulties and negative self-image. These findings align with those of Reuter et al. (2022), who demonstrated that transitions from employment to unemployment during the STWT process can adversely affect young people’s health and well-being [6]. For some participants, this meant trying different jobs to identify work that was suitable, considering their health and OCD. Workplace stressors often made it difficult to initiate tasks and maintain focus because of obsessions and compulsive rituals. However, for some participants, work could also be a distraction that reduced the impact of these symptoms. Supportive workplace relationships, focusing on strengths rather than difficulties, and healthy working conditions with opportunities for adequate recovery were all described as promoting and enhancing participants’ ability to remain in employment.

An earlier realist study illustrated how the interrelation between personal and work-related factors influences opportunities for work participation among employees with common mental health problems [33]. The study also highlighted the importance of different types of social support in the workplace [33]. Efforts to support entry into work, described in our study, included flexible working hours, less stressful job roles, and the opportunity to clarify and structure work tasks that elicited feelings of uncertainty.

Neal-Barnett and Mendelson (2003) examined the role of OCD in the workplace among women, suggesting several strategies to promote work participation: allowing frequent breaks during the working day, providing a workplace mentor, and adopting a holistic view of the individual, rather than focusing solely on the diagnosis of OCD [18]. The authors emphasised that accommodations should be individually tailored and based on collaboration and dialogue with the individual in question [18]. These accommodations may also be crucial for young adults with OCD entering the labour market; however, this requires that managers are aware of the individual’s diagnosis and associated difficulties. These studies, as well as our findings, highlight the importance of a supportive work environment. This may be a crucial factor in enabling young adults with OCD to enter and remain in employment, while also supporting personal development and strengthening self‑image.

The findings show that participants had developed various strategies and sought support to manage challenges, navigate studies or transitions into work, and maintain health and well-being. Social support from friends, family or partners was central and was described as helpful, in managing the STWT process and alleviating stress and OCD symptoms. A study by Palardy et al. (2018) similarly found that positive social support may be beneficial for individuals with OCD [34]. Strategies used included maintaining daily routines, planning and establishing structure, which facilitated participation in both studies and work. To balance demands, protect health and restore energy, participants used different strategies, such as allowing time for recovery and doing things that made them feel good. For example, creative activities were reported to reduce OCD symptoms and anxiety, and physical activity was described as alleviating anxiety during stressful periods and improving focus.

A previous systematic review and meta-analysis showed that various forms of exercise, e.g. aerobic exercise and yoga, were associated with reduced symptoms of OCD, depression and anxiety. The authors therefore proposed that physical exercise may serve as a potential intervention to improve OCD symptoms, as well as depression and anxiety [35]. In addition, examples of strategies used to overcome challenges and maintain or balance health may be valuable for healthcare professionals and other stakeholders working with this group, and could be conveyed to young adults with OCD, as these strategies may be useful in everyday life and during the STWT process.

This study suggests that societal support needs to be tailored to the individual to optimise the STWT process and prevent delays or setbacks. Managing contacts with different organisations, e.g. the SIA and the PES, and a lack of communication between agencies, were experienced as challenging by participants with OCD. Being referred within and between organisations, as well as having to repeatedly explain one’s situation, was perceived as stressful and could negatively affect health. Stress related to contact with different agencies has also been confirmed in previous research [36], as has the importance of individual adaptation in vocational rehabilitation [36, 37], although not specifically among young adults with OCD. To improve conditions for young adults with OCD, and likely also others with mental disorders, in the STWT process, organisational structures and working practices need to be reviewed and adapted to better address individual needs.

The role of healthcare was described as valuable for facilitating participation in studies and entry into the workforce and included counselling, psychological, and/or medical treatment. In Sweden, coordination efforts should be provided to patients on sick leave, when needed, to support their return-to-work or entry into working life [38]. These efforts are usually performed by a rehabilitation coordinator, whose role comprises three main areas: coordination within the healthcare system; providing individually tailored support to patients; and collaboration with external actors [38], for example, the SIA, PES, prospective employers and municipalities involved in the vocational rehabilitation.

The contribution of rehabilitation coordinators to patients in psychiatric care has been described by healthcare professionals as increasing the likelihood of identifying suitable occupations that match individual needs, as their work is grounded in a holistic approach. This means taking into account individuals’ needs, e.g. recovery, when planning the process of entry into work/studies [39]. Such support has been described as especially important for young people, in order to prevent negative health effects [39]. The challenges described by participants in our study indicate that rehabilitation coordination may represent a valuable opportunity to support young adults with OCD, or other mental disorders, during the STWT process. Interventions involving rehabilitation coordination to support the STWT process in young adults with mental disorders, such as OCD, may represent a priority area and provide a basis for further research.

The findings also highlight that public stigma, along with limited knowledge and understanding of OCD and mental illness in society, can act as barriers to disclosure. Consequently, several participants lived with their OCD diagnosis in secrecy, sharing it only with their closest ones. Fear of being seen in a particular way or feeling ashamed were reported as reasons for non-disclosure.

Similar findings have been reported by Keyes et al. (2018) [40]. Participants described weighing the pros and cons of disclosure, although disclosure was perceived to increase the likelihood of receiving support or accommodations to facilitate study engagement or entry into work, which some had experienced. However, there was also a fear of being rejected or replaced by someone if the diagnosis was disclosed during the job application or hiring process. Similar findings were reported in a review by Lindsay et al. (2017), where young adults with disabilities were reluctant to disclose their diagnosis during recruitment or the probationary period but felt more comfortable doing so afterwards [41].

One of the key strengths of this study is the use of in-depth interviews with young adults living with OCD who were at different stages of the STWT process. The diversity among participants in terms of personal characteristics and experiences contributed to rich data. Participants included both men and women, with varying ages (range 19–28 years), educational levels and geographic regions. The richness of the data, confirmed by the participants’ quotations, enhanced credibility [29, 30].

Dependability was enhanced by using the same interview guide for all participants. Another strength is that the interviews provided information on both challenging and supportive factors within the STWT process from individual, organisational, and societal perspectives, thereby enabling an exploration of participants’ experiences across different contexts. This knowledge can contribute to a better understanding of the challenges that young people with OCD may face during the STWT process, as well as how interventions can address both individual needs and different environmental circumstances, thereby enhancing opportunities for inclusion in working life and participation in society. Moreover, the research team contributed to reflexive discussions throughout the research process, which constitutes an additional indicator of quality [42]. To the best of our knowledge, this topic has not previously been studied. Moreover, the detailed, transparent description of the context, participant recruitment, and research process enables readers to assess the transferability of the findings to other settings and contexts [43].

The study has several limitations. First, a higher proportion of participants were female. There is also a risk of selection bias, as most participants were recruited through social media; consequently, individuals who do not use social media may not have been reached by the study. It is also possible that young adults with severe OCD symptoms, for example, due to functional difficulties or stigmatisation, were less likely to participate. Therefore, caution is warranted when interpreting the findings, as the severity of OCD symptoms and their potential impact on functioning may have influenced participation. Another potential limitation of the study is that the reference group was not involved in the analysis of the interview data. Involving members of the reference group in the analysis may have identified additional aspects of the data that were not apparent to the researchers.

## Conclusion

The study shows how young adults with OCD navigate STWT process through various experiences of education, employment and unemployment, while also trying to maintain their mental health and well-being. The findings may suggest a potential interplay between OCD symptoms and engagement in education and employment during this period, implying that aggravating OCD symptoms or worsening overall mental health in challenging situations could contribute to delays or interruptions in the STWT process. Delays or interruptions were also evident when there was a dissonance between available support and individuals’ needs. In contrast, pedagogical or workplace accommodations, social and societal support, along with the development of one’s own coping strategies may help young people navigate this complex transitional period.

The implications of this study suggest booth a need for flexible, individually tailored support for this vulnerable group and for coordinated efforts among relevant stakeholders. Healthcare interventions may extend beyond managing symptoms and daily functioning to also address the new contexts and situations encountered by young adults. Overall, increased awareness of OCD, and sustainable and supportive study and work environments, is likely important for facilitating the STWT process among young adults with mental health disorders, such as OCD.

## Supplementary Information

Below is the link to the electronic supplementary material.

Supplementary Material 1

## Data Availability

The datasets generated and analysed during the current study are not publicly available due to the Swedish ethical review regulations. Data may be available from the corresponding institution upon reasonable request. Requests for access should be directed to Uppsala University, Department of Public Health and Caring Sciences, Public Health, Working Life and Rehabilitation, Box 256, 751 05 Uppsala, or to the principal investigator, Åsa Andersén, (asa.andersen@uu.se), who may then seek permission from the Swedish Ethical Review Authority.

## Declarations

### Use of artificial intelligence

Grammarly (licensed version) was used for language improvement. In addition, Microsoft Copilot (secure, university‑licensed version) was used to assist with the translation of individual words and the language refinement of selected sentences during manuscript preparation. Copilot was also used to provide suggestions for condensing the conclusion section and improving the conciseness. All suggestions were reviewed, edited, and approved by the authors, who take full responsibility for the final content of the manuscript.

## Abbreviations

OCD: Obsessive-compulsive disorder
PES: Public employment service
SIA: Social insurance agency
STWT: School-to-work or school-to-university transition
