# Supplementary material for: Young adults with OCD and their experiences of the school-to-work or university transition: an explorative qualitative study
Source: BMC Psychiatry. 2026 Oct 13;26:826. doi: 10.1186/s12888-026-08714-6 (PMC13711837; doi:10.1186/s12888-026-08714-6)
Supplement: Supplementary file 1 [file 12888_2026_8714_MOESM1_ESM.docx]

**Supplementary file 1. Interview guide**

The interview guide is semi-structured, allowing flexibility, follow-up questions and prompts. Questions or headings in bold mark the interview areas and indicate where the researcher aimed to elicit an in-depth account of the participant’s experiences and situation.

**Would you like to start by telling me a bit about yourself?**

Where are you in life right now?

What does a typical day look like currently? (Interest in the person’s daily life and context)

- Tell me about when you were diagnosed with OCD.
- How does OCD manifest itself for you in your daily life? (thoughts, behaviours)

**What are your thoughts about your upper secondary education period?**

- How did a typical day look like for you during school? (routines before-, during and after school time; includes both schoolwork and free time)
- Was there anything or anyone during your upper secondary education period that made your studies easier?
- Questions about structural or individual support at school or privately, e.g. healthcare services, student health services, family, friends (what and how, what did it mean? Examples of situations)
- Was there anything that made it difficult to study? (structural, individual or private)
- How did OCD symptoms affect your schooling? (routines, risk of not being able to move on in activities, stigma, other)
  - Were you able to be open about your symptoms?
- Were you able to complete your upper secondary education with full grades?
  - If not, how do you view finishing your upper secondary education without full grades?
- Did you receive any support related to OCD during your upper secondary education period?

**What expectations did you have about working or studying after your upper secondary education period?**

(expectations regarding the transition from upper secondary education to entry into post-secondary education or work, outcomes and current status).

- Can you describe how you experienced others’ expectations?
- Can you describe how you think about working?
- How did/do you view your opportunities to work after upper secondary education?
- From a societal perspective (societal conditions, labour market, opportunities for participation)
- How did you view your personal circumstances?
- Did you feel that you had the opportunity to choose your path after upper secondary education? Can you elaborate? (individual perspective, personal conditions, preparation)
- What was it like for you when you finished upper secondary education? Can you describe what your daily life looked like at that time?

**What did your participation in work or studies look like after** **upper secondary education?** (personal circumstances)

- Were there other activities you took part in?
- Where are you now?
- If you are working/studying, can you describe how it is going?
- What was it like for you—was there anything you did to make it easier to start working/studying with regard to OCD?
  - Choice of work/studies?
- Was/is there anything about you that made/makes it difficult to start working or studying? (autonomy, agency, experience of doing)

**Environmental and contextual aspects**

If you think about your environment, was/is there someone/something that made it easier for you in starting work or studies?

- Looking at societal support, is there anything that has facilitated entry into work or studies?
- Can you describe the support you received? (support related to symptom relief/work-oriented support)
  - Public Employment Service, Social Insurance Agency, employer, healthcare services, structural support for inclusion, guidance counsellor, other?
  - What did/do these contacts look like?
- If you think about your surroundings, was/is there anyone who was/is a source of support for you in starting work or studies?
- Was/is there anything in your societal environment that makes it difficult to work or study? (interest in norms, stigma, structural support)
- From your perspective, what would optimal support look like/have looked like with regard to OCD?

**How do you view establishing yourself in work in the future?**

- Goals?
- Anything else?
